# Prioritizing radiotherapy infrastructure in depopulating societies: a 2050 blueprint from Akita, Japan

**DOI:** 10.1093/jrr/rrag033

**Published:** 2026-05-14

**Authors:** Yuhei Koike, Satoaki Nakamura, Yuki Wada

**Affiliations:** Department of Radiology, Kansai Medical University, 2-5-1 Shinmachi, Hirakata, Osaka 573-1010, Japan; Division of Radiation Oncology, Kansai Medical University Hospital, 2-3-1 Shinmachi, Hirakata, Osaka 573-1191, Japan; Department of Radiology, Kansai Medical University, 2-5-1 Shinmachi, Hirakata, Osaka 573-1010, Japan; Division of Radiation Oncology, Kansai Medical University Hospital, 2-3-1 Shinmachi, Hirakata, Osaka 573-1191, Japan; Department of Radiology, Akita University Graduate School of Medicine, 1-1-1 Hondo, Akita, Akita 010-8543, Japan

**Keywords:** geographic access, radiotherapy, IMRT, facility location, p-median, population projection

## Abstract

In super-aged and depopulating societies, repeated travel requirements for radiotherapy (RT) can amplify geographic inequities in access to advanced techniques. The aim of this study was to determine optimal placement of intensity-modulated radiotherapy (IMRT)-capable facilities in Akita Prefecture—a leading-edge case of Japan's demographic transition—using p-median optimization under population projections through 2050. Using 1-km mesh population data for 2020 and official projections for 2030, 2040 and 2050, we calculated one-way automobile travel times from each mesh centroid to RT facilities with the Open Source Routing Machine based on OpenStreetMap road data. A 60-min travel-time threshold defined access coverage. IMRT-capable facilities were identified from official insurance-designation lists. Optimal placement was determined using a population-weighted p-median model, with the number of facilities prespecified as one per 200 000 population. In 2020, 10 external-beam RT facilities provided near-universal 60-min coverage (99.99%; mean 12.8 min), whereas the single IMRT-capable facility covered only 70.3% (uncovered 285 273; mean 42.5 min). Optimized placement with five IMRT-capable facilities increased coverage to 99.99% (uncovered 89) and reduced mean travel time to 14.2 min. Under population projections, optimized placement maintained >99% coverage through 2050 with three facilities, and three core locations were consistently selected across all time horizons. These temporally stable core locations can serve as priority targets for long-term IMRT infrastructure investment in aging and depopulating regions.

## INTRODUCTION

Radiotherapy (RT) requires daily attendance over several weeks, making travel burden a practical determinant of treatment initiation, adherence and completion. This issue is becoming more consequential in super-aged and depopulating regions, where a growing share of patients have limited mobility and where specialized services tend to concentrate in fewer centers [1–3].

Intensity-modulated radiotherapy (IMRT) has become a standard technique for multiple malignancies because it enables conformal dose escalation while reducing exposure to surrounding normal tissues. At the same time, IMRT depends on specialized equipment and expertise, which encourages centralization. This creates a recurrent planning trade-off: concentrating services to maintain quality and efficiency versus distributing services to preserve geographic equity.

Japan provides a useful setting to examine this trade-off. Despite a well-established national RT infrastructure [4], IMRT capability remains unevenly distributed and is concentrated in large hospitals, resulting in marked geographic disparities—especially in rural areas [5–7]. This concentration is partly attributable to the facility accreditation requirements under Japan's national health insurance reimbursement system, which impose specific staffing and equipment standards that facilities must meet to claim reimbursement for IMRT [8]. These requirements, which are relatively unusual by international standards, effectively restrict IMRT provision to larger, well-resourced institutions, limiting geographic availability particularly in rural areas. While these regulatory requirements are specific to Japan, geographic disparities in access to RT are a broader concern. Longer travel distance or time to external-beam RT (EBRT) facilities has been associated with lower utilization in multiple settings [2, 3, 9]. Moreover, distance-related barriers appear more consequential among older patients, underscoring the clinical relevance of geographic access in aging societies [10].

Several studies have applied spatial optimization to EBRT facility planning. For example, p-median and location-allocation approaches have been used to evaluate the geographic distribution of EBRT centers in Belgium, and strategic placement studies in Canada have demonstrated that adding new facilities can reduce the population with poor access [11, 12]. More recently, optimization work in France has examined strategic placement and reallocation of RT units to improve accessibility [13]. However, an important limitation is that these studies optimized facility placement at a single time point and did not incorporate future population projections [11–13].

In societies undergoing simultaneous population aging and sustained population decline—a trajectory now shared by Japan, parts of Southern and Eastern Europe and East Asia—facility configurations that are optimal today may not remain optimal over the coming decades [14]. This gap is particularly consequential for high-cost, expertise-intensive services such as IMRT, where planning must balance centralization to maintain quality and efficiency against decentralization to preserve geographic equity over time.

Akita Prefecture provides an advanced case of Japan’s demographic transition. Based on Japan’s official Population Estimates, Akita had the highest proportion of residents aged ≥65 years in 2024 (39.5%) [15]. According to the latest regional population projections, this proportion is expected to increase further to 49.9% by 2050, while the total population is projected to decline from 960 000 in 2020 to 560 000 in 2050 (approximately −42%) [16]. The aim of this study was to determine the optimal placement of IMRT-capable facilities in Akita Prefecture using a p-median optimization framework under multi-temporal population projections (2020, 2030, 2040 and 2050). Unlike prior single-time-point optimization studies, we further sought to identify temporally stable ‘core’ locations that remain optimal across a 30-year horizon, thereby providing a practical framework to prioritize long-term infrastructure investment under demographic uncertainty.

## MATERIALS AND METHODS

### Study design

This was a simulation study using mathematical optimization to identify optimal locations for IMRT-capable facilities in Akita Prefecture, Japan. Akita Prefecture was selected as the study area because it has the highest aging rate in Japan (37% of the population aged 65 years or older) and is experiencing substantial population decline. The demographic profile of Akita Prefecture represents the projected future trajectory of many other Japanese regions, making it a suitable model area for facility planning studies. The analysis was conducted for four time points: 2020 (baseline), 2030, 2040 and 2050.

### Data sources

Population data at the 1-km mesh level were obtained from the National Land Numerical Information database, published by the Ministry of Land, Infrastructure, Transport and Tourism of Japan. This dataset includes both the 2020 baseline population and projected populations for 2030, 2040 and 2050. The locations of existing EBRT facilities were compiled from publicly available hospital information and verified manually, as described in our previous study [5]. IMRT-capable facilities were identified using the official insurance-designation lists published by the Regional Bureaus of Health and Welfare of the Ministry of Health, Labour and Welfare; facilities were classified as IMRT-capable if they were registered as insured medical institutions authorized to claim reimbursement for IMRT. As of 2020, Akita Prefecture had ten EBRT facilities, of which one was capable of delivering IMRT. Road network data were obtained from OpenStreetMap (OSM), which provides detailed road geometry and attributes for routing calculations.

### Travel time calculation

Travel times from each 1-km mesh centroid to all existing EBRT facilities and to candidate locations for IMRT placement were calculated using the Open Source Routing Machine (OSRM), a routing engine based on OSM data snapshot dated 28 August 2025 [17]. Congestion and time-of-day effects were not modeled, yielding free-flow travel-time estimates. For each mesh, the travel time to the nearest facility was recorded.

### Optimal facility placement

#### Access threshold

A 60-min one-way travel time was used as the threshold for geographic access. This threshold was based on prior studies indicating that 92.4% of the United States population can access EBRT facilities within 60 min [18], and that travel times exceeding 60 min are associated with increased healthcare costs and hospitalization rates [10].

#### Number of facilities

The number of IMRT-capable facilities to be placed was prespecified as a planning scenario for access optimization rather than being estimated from detailed service throughput. Because IMRT is typically delivered on megavoltage linear accelerators, we referenced international RT capacity planning benchmarks expressed as megavoltage units per million population. The ESTRO-QUARTS project estimated an average requirement of 5.9 megavoltage units per million population (≈ one unit per ~170 000 population), and the IAEA planning tool reports guideline values of a similar order (e.g. approximately one linear accelerator per ~180 000 persons) [19, 20]. In line with this order-of-magnitude benchmark, we operationalized the number of IMRT-capable facilities as one facility per 200 000 population (rounded up), treating this as a pragmatic approximation under population decline.

#### Optimization model

The p-median model [21] was used to identify optimal facility locations. The objective was to minimize the total population-weighted travel time:


$$ \mathit{\min}\sum_{i=1}^n\sum_{j=1}^m{w}_i{d}_{ij}{x}_{ij} $$

where *n* is the number of demand points, *m* is the number of candidate locations, *w_i_* is the population of demand point *i*, *d_ij_* is the travel time from demand point *i* to candidate location *j*, and *x_ij_* is a binary variable equal to 1 if demand point *i* is assigned to facility *j* and 0 otherwise. Each demand point was assigned to its nearest open facility, and the total number of IMRT-capable facilities was constrained to *p*. Candidate IMRT-capable facility locations were defined as the centroids of all 1-km mesh cells with non-zero population. The optimization was solved using a greedy heuristic algorithm. At each iteration, the algorithm selected the candidate location that produced the greatest reduction in total population-weighted travel time, continuing until the target number of facilities was reached.

Three outcome measures were calculated for each scenario. The coverage rate was defined as the percentage of the population within 60 min of the nearest IMRT-capable facility. The uncovered population was the number of individuals with travel times exceeding 60 min. The population-weighted mean travel time was calculated as the average travel time weighted by mesh population.

## RESULTS

**Fig. 1:**
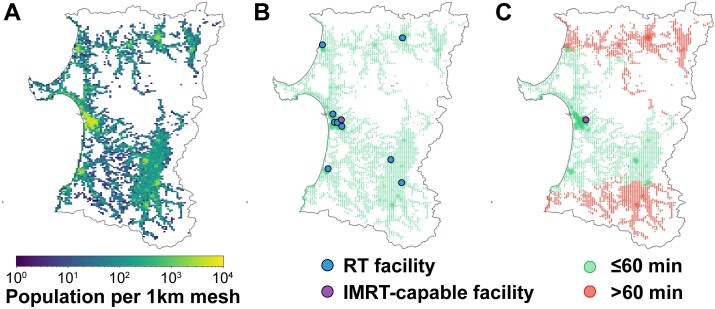
Geographic access to RT facilities in Akita Prefecture in 2020. (A) Population distribution per 1-km mesh. (B) Coverage of RT facilities. (C) Coverage of IMRT-capable facilities. The purple circle indicates the single IMRT-capable facility. Green and red areas represent populations within and beyond 60 min, respectively.

Figure 1 shows the geographic access to current EBRT and IMRT-capable facilities in Akita Prefecture in 2020. The population of Akita Prefecture was 959 501, concentrated primarily in Akita City in the central-western region, with additional clusters in Odate and Noshiro cities in the north, and Yokote, Daisen and Yurihonjo cities in the south (Fig. 1A). All ten existing RT facilities, including the single IMRT-capable facility, were distributed in alignment with these population centers, and 99.99% of the population could access a facility within 60 min (Fig. 1B). The mean travel time was 12.8 min. In contrast, only one IMRT-capable facility existed in the prefecture. The 60-min coverage for IMRT was 70.3%, leaving 285 273 individuals without access within the threshold (Fig. 1C). The mean travel time to the nearest IMRT-capable facility was 42.5 min. Table 1 summarizes the population, facility count, coverage and travel time under current and optimal configurations for each year.

**Table 1:** Coverage comparison between current and optimal IMRT facility placement

		Current				Optimal			
Year	Population	Facilities (*n*)	Coverage (%)	Uncovered (*n*)	Mean travel time (min)^a^	Facilities (*n*)^b^	Coverage (%)	Uncovered (*n*)	Mean travel time (min)^a^
2020	959 501	1	70.3	285 273	42.5	5	99.99	89	14.2
2030	818 711	1	71.6	232 272	41.2	5	99.99	80	13.8
2040	686 200	1	72.9	185 948	40.0	4	99.99	70	15.1
2050	560 428	1	74.3	144 053	38.7	3	99.89	633	17.1

^a^Population-weighted mean travel time to the nearest facility. ^b^Calculated as population divided by 200 000 (rounded up).

**Fig. 2:**
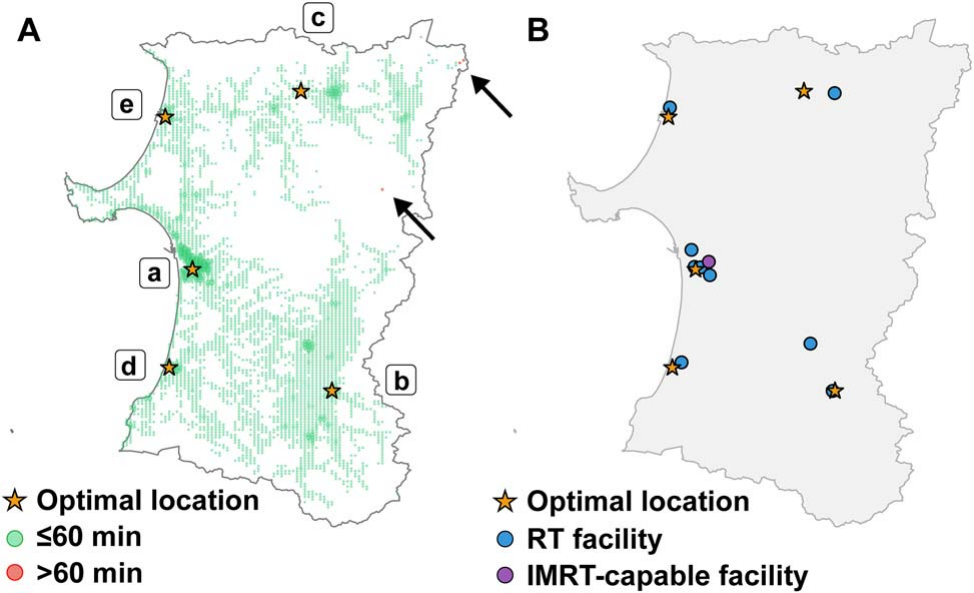
Optimal placement of IMRT facilities in Akita Prefecture in 2020. (A) Coverage under optimal placement with five facilities (stars, labeled A-E). Covered (≤60 min) and uncovered (>60 min) populations are distinguished by shading. Arrows indicate areas remaining outside the threshold. (B) Spatial relationship between optimal locations, existing RT facilities.

Figure 2 summarize the results of optimal IMRT-capable facility placement (see Supplementary Table 1 for specific coordinates). Based on the criterion of one facility per 200 000 population, the required number of IMRT-capable facilities for 2020 was calculated as five. The p-median optimization identified five locations that achieved 99.99% coverage within 60 min (Fig. 2A). The uncovered population decreased from 285 273 to 89, and the mean travel time decreased from 42.5 to 14.2 min (Table 1). The spatial relationship between the optimal locations and existing facilities is shown in Fig. 2B. Arrows in Fig. 2A indicate areas that remained outside the 60-min threshold.

**Fig. 3:**
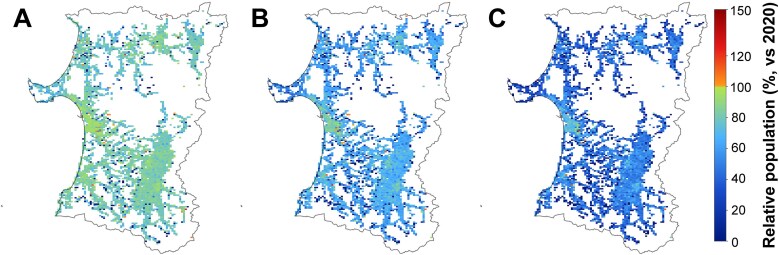
Projected population change in Akita Prefecture relative to 2020. (A) 2030, (B) 2040 (C) 2050. Color scale indicates relative population compared to 2020, where green represents maintained population and red/orange indicates decline. Population projection data were obtained from the National Land Numerical Information database (Ministry of Land, Infrastructure, Transport and Tourism of Japan).

Figure 3 shows the projected population change in Akita Prefecture at the 1-km mesh level for 2030 (A), 2040 (B) and 2050 (C). The population is projected to decrease from 959 501 in 2020 to 560 428 in 2050, a 42% reduction (Table 1). Population decline was observed across most areas, with particularly pronounced decreases in rural regions. These patterns are projected to occur in many regions throughout Japan (see Supplementary Fig. 1).

**Fig. 4:**
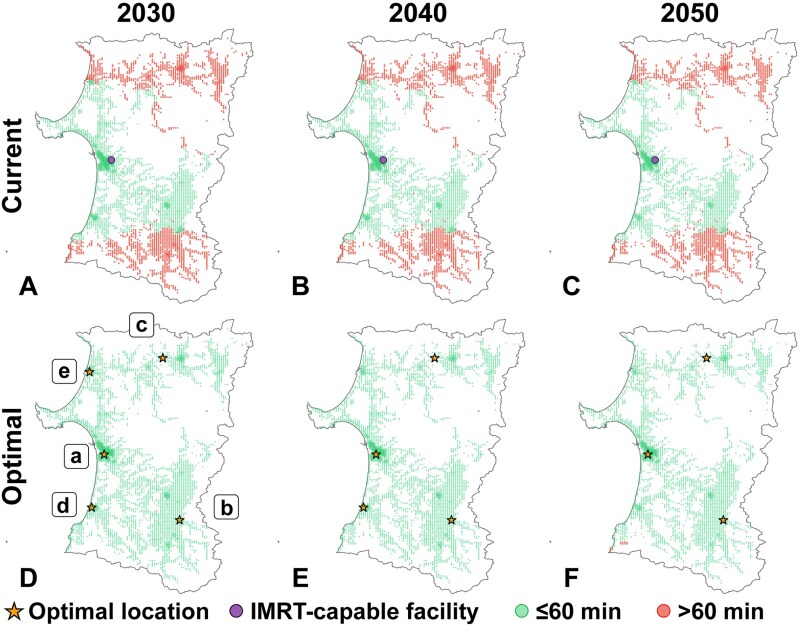
Comparison of IMRT coverage under current and optimal facility placement for future years. Upper row (A–C): coverage with the current single IMRT-capable facility for 2030, 2040 and 2050. Lower row (D–F): coverage with optimal facility placement for 2030 (five facilities), 2040 (four facilities) and 2050 (three facilities). Covered (≤60 min) and uncovered (>60 min) populations are distinguished by shading.

Figure 4 compares coverage under the current single IMRT-capable facility (A–C) and optimal placement (D–F) for 2030, 2040 and 2050. Based on the population criterion, the required number of facilities decreased from five (2020–30) to four (2040) and three (2050) as the population declined. Under the current configuration, coverage remained between 71.6 and 74.3%. Under optimal placement, coverage was maintained at >99% across all time points (Table 1). Three locations (a, b and c) were consistently selected across all four time points.

## DISCUSSION

This study revealed a substantial disparity in geographic access to RT services in Akita Prefecture, which is one of the most rapidly depopulating prefectures in Japan and represents an advanced case of the demographic challenges confronting aging and shrinking societies worldwide, including parts of Southern and Eastern Europe and East Asia. While the existing ten photon-capable facilities provided near-universal coverage (>99% within 60 min), the single IMRT-capable facility covered only 70.3%, leaving ~285 000 residents beyond the 60-min threshold. Using p-median optimization, we showed that strategically placing five IMRT-capable facilities could achieve 99.99% 60-min coverage, substantially reducing the geographic access gap.

The present study contributes to the facility-planning literature in three ways. First, rather than evaluating access under existing configurations alone, we applied mathematical optimization to identify where IMRT-capable facilities should be located to minimize population-weighted travel time and improve coverage. Second, by incorporating population projections through 2050, we framed facility planning as a multi-temporal problem in which the appropriate configuration may shift as demand changes under sustained depopulation. Third, we identified temporally stable ‘core’ locations that were consistently selected across all time horizons, providing quantitative evidence to prioritize long-term investment under demographic uncertainty.

Geographic barriers to RT have clinically and economically relevant consequences. Travel burden has been widely reported as a barrier across the cancer care continuum, including delayed diagnosis, reduced treatment receipt and poorer outcomes [22]. In older Medicare beneficiaries with cancer, travel times longer than 1 h were associated with 14% higher Medicare spending during the initial phase of care and with higher hospitalization rates, compared with travel times of 30 min or less [10]. Given that RT typically requires daily attendance for 4–7 weeks, the cumulative travel burden directly affects treatment initiation, adherence and completion. Importantly, the impact of distance-related barriers appears to be amplified in older populations; Pagano *et al.* demonstrated in Italy that the reduction in RT utilization associated with distance was more pronounced among patients aged 70 and older [23]. As the proportion of older patients rises in aging societies, optimizing geographic access to RT becomes an increasingly important component of equitable cancer care.

Akita Prefecture is projected to undergo a substantial population decline, from 960 000 in 2020 to 560 000 by 2050 (Fig. 3), directly affecting the required number of IMRT facilities. Consistent with this demographic shift, the scenario-based number of facilities decreased from five in 2020 to three in 2050, illustrating how an access-oriented configuration can adapt to changing demand. Notably, three core locations (locations a, b and c in Fig. 4) were consistently selected across all time points, suggesting that these sites are robust candidates for long-term investment. As illustrated in Fig. 4, while the current single IMRT facility maintains coverage at only 70–74% throughout the projection period, the optimal placement sustains coverage above 99% even in 2050 with just three facilities. These temporally stable core locations have practical implications: investments directed at these sites are likely to remain effective regardless of how population dynamics unfold, balancing the need to avoid overinvestment while ensuring adequate access.

The optimal locations identified by the model do not perfectly coincide with existing photon-capable facilities; however, as shown in Fig. 2B, several optimal locations are situated in close proximity to existing facilities. This suggests that upgrading existing facilities by adding IMRT capabilities may represent a more realistic and cost-effective strategy than constructing new facilities. This methodology is intended to provide reference information for policy decision-making rather than to prescribe a definitive configuration; actual decisions would require consideration of treatment capacity, availability of specialized personnel, equipment compatibility and financial feasibility. It is important to note that areas not selected as optimal IMRT locations, such as those currently served by existing photon-capable facilities, would retain an essential role in the regional RT network. These facilities would continue to provide conventional and palliative RT services, which are particularly important for patients with limited mobility or poor performance status for whom even a 60-min travel time may impose an unacceptable burden. The present optimization identifies candidate locations for adding IMRT capabilities and is not intended to suggest reduction or closure of existing services.

The demographic challenge addressed in this study extends well beyond Japan. Spatial optimization approaches have been applied to RT facility planning in several countries, including Belgium [11], Canada [24] and France [13]; however, all of these studies optimized facility placement at a single time point. By incorporating multi-temporal population projections, the present study identifies core locations that remain optimal across a 30-year horizon, offering a framework directly applicable to any region facing sustained population decline. By 2050, many regions across Japan will exhibit population patterns similar to present-day Akita (see Supplementary Fig. 1), and comparable demographic trajectories are projected in Southern and Eastern Europe and East Asia.

Several limitations should be acknowledged. First, the analysis was conducted in a single prefecture, and extrapolation to regions with different characteristics requires validation. Second, the number of IMRT-capable facilities was determined using a fixed population ratio that does not dynamically adapt to regional conditions, and the model did not account for facility capacity or availability of specialized personnel. Third, only automobile travel times were considered, and public transportation access was not evaluated. The travel times calculated by OSRM do not account for real-world conditions such as traffic congestion, traffic signals, or seasonal road closures due to snowfall, potentially underestimating actual travel times. Fourth, the analysis relies on several assumptions about the future. Population projections carry inherent uncertainty; the current facility accreditation requirements for IMRT were assumed to remain unchanged despite the possibility of future technological innovations or regulatory relaxation, and total population was used as a proxy for RT demand. Because cancer predominantly affects older adults, age-adjusted cancer incidence rates would provide a more appropriate measure of RT demand than total population. Future studies incorporating age-specific projections would provide more refined demand estimates. Despite these limitations, the present study benefits from Japan's nationally standardized 1-km mesh population data with built-in future projections, providing a spatial granularity rarely available in other countries. The methodology itself is transferable to any setting with comparable population projection data, facility locations and road network information, and validation in regions with different geographic and demographic characteristics would be a valuable next step.

In conclusion, this study demonstrated that while existing EBRT facilities in Akita Prefecture provide near-universal geographic coverage, IMRT capabilities remain insufficient and geographically concentrated, leaving ~30% of the population beyond the 60-min access threshold. p-median optimization demonstrated that strategic placement of IMRT facilities could dramatically improve geographic coverage, and that the optimal configuration can adapt to projected population decline while sustaining high coverage levels. Three core locations remained consistently selected across all time horizons, offering robust targets for long-term investment. Several of these optimal locations are proximate to existing EBRT facilities, suggesting that functional upgrades may represent a feasible implementation pathway. This methodology provides quantitative reference information for policy decisions aimed at the efficient allocation of limited healthcare resources in aging and depopulating societies.

## Supplementary Material

SupplementaryData_rrag033

## Presentation at a conference

None declared.
