# Supplementary material for: Prioritizing radiotherapy infrastructure in depopulating societies: a 2050 blueprint from Akita, Japan
Source: J Radiat Res. 2026 May 14;67(4):584–90. doi: 10.1093/jrr/rrag033 (PMC13400574; doi:10.1093/jrr/rrag033)
Supplement: SupplementaryData_rrag033 [file supplementarydata_rrag033.docx]

**Supplementary Figure**

| 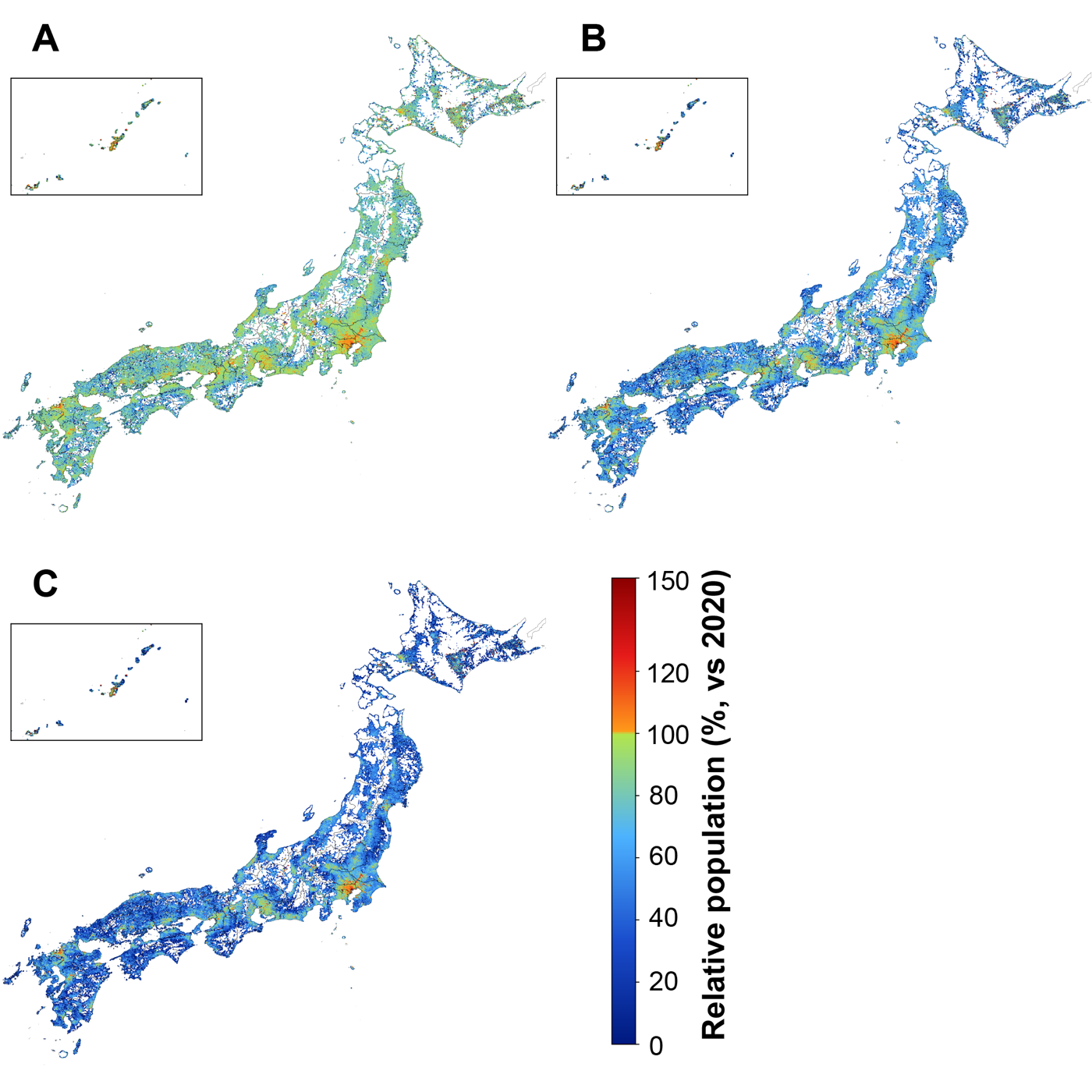 |
| --- |
| **Fig 1.** Projected population change across Japan relative to 2020. (A) 2030, (B) 2040, (C) 2050. Color scale indicates relative population compared to 2020. By 2050, widespread population decline is projected across rural and mountainous regions throughout Japan, while population growth is largely confined to major metropolitan areas such as Tokyo. These patterns indicate that the geographic access challenges identified in Akita Prefecture are likely to emerge in many other regions facing similar demographic trends. Population projection data were obtained from the National Land Numerical Information database (Ministry of Land, Infrastructure, Transport and Tourism of Japan) |

**Supplementary Table**

**Table 1.** Optimal facility locations based on the p-median optimization model in 2020

| Location | Latitude | Longitude |
| --- | --- | --- |
| a | 39.704 | 140.106 |
| b | 39.312 | 140.556 |
| c | 40.279 | 140.456 |
| d | 39.388 | 140.031 |
| e | 40.196 | 140.019 |
